# A route to decreasing N pollution from livestock: Use of *Festulolium* hybrids improves efficiency of N flows in rumen simulation fermenters

**DOI:** 10.1002/fes3.209

**Published:** 2020-05-22

**Authors:** Stephen Kamau, Alejandro Belanche, Teri Davies, Pauline Rees Stevens, Mike Humphreys, Alison H. Kingston‐Smith

**Affiliations:** ^1^ Institute of Biological, Environmental and Rural Sciences (IBERS) Aberystwyth University Aberystwyth UK; ^2^ Estacion Experimental del Zaidín (CSIC) Granada Spain

**Keywords:** *Festuca*, *Festulolium*, *Lolium*, nitrogen‐use efficiency, protein, proteolysis, rumen

## Abstract

Ruminant agriculture suffers from inefficient capture of forage protein and consequential release of N pollutants to land. This is due to proteolysis in the rumen catalyzed by both microbial but initially endogenous plant proteases. Plant breeding‐based solutions are sought to minimize these negative environmental impacts. The aim of this study was to perform an integrated study of rumen N metabolism using semi‐continuous rumen simulation fermenters (Rusitec) to explore the extent to which swards containing *Festulolium* populations (interspecific hybrids between *Lolium* and *Festuca* grass species) with decreased rates of endogenous protein degradation conferred advantageous protein utilization in comparison with a National Listed perennial ryegrass. An in vitro experiment was conducted using three *Festulolium* hybrids (*Lolium perenne* × *Festuca arundinacea var. glaucescens*, LpFg; *Lolium perenne* × *Festuca mairei*, LpFm; and *Lolium multiflorum* × *Festuca arundinacea var. glaucescens*, LmFg) and a *Lolium perenne*, Lp control. LpFm and LmFg demonstrated significantly lower plant‐mediated proteolysis than the control. Fresh forage was incubated in Rusitec with rumen fluid from four donor cows. Feed disappearance and production of gas, methane, and volatile fatty acids were similar across cultivars. Whereas no differences in microbial protein synthesis were noted across treatments during early fermentation (0–6 hr after feeding), an increased microbial N flow in LpFm (+30%) and LmFg hybrids (+41%) was observed during late fermentation (6–24 hr after feeding), with higher overall microbial N flows (+13.5% and + 20.2%, respectively) compared with the control (Lp). We propose an underpinning mechanism involving the partitioning of amino acid catabolism toward branched‐chain amino acids and microbial protein synthesis in grasses with slow plant‐mediated proteolysis instead of accumulation of rumen ammonia in grasses with fast plant‐mediated proteolysis. These observations indicate the potential of *Festulolium* hybrids with a slow plant‐mediated proteolysis trait to improve the efficiency of capture of forage protein and decrease the release of N pollutants onto the land.

## Graphical abstract

Excessive protein loss from fresh forage feeds is a problem for ruminant agriculture. Festulolium hybrids identified with lower endogenous induced proteolysis in response to rumen conditions also demonstrated improved N flows in rumen‐simulating fermenters and decreased ammonia production with no negative impact on microbial protein synthesis. These hybrids offer considerable potential for decreasing the environmental impact of ruminant agriculture.

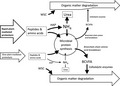

## 1 INTRODUCTION

Ryegrass (*Lolium* spp*.*) is often the forage grass of choice in mild temperate climates due to its high yield and nutritious value (Capstaff & Miller, 2018; Humphreys, Yadav, et al., 2006). However, milk production is limited by lower crude protein content and dry matter intake compared with other forages such as legumes (Dewhurst, Fisher, Tweed, & Wilkins, 2003b). Furthermore, inefficient capture of forage N by ruminants is a significant problem for livestock farming, resulting in the need for protein supplementation and consequential release of nitrogenous pollutants to the environment (Dewhurst, Mitton, Offer, & Thomas, 1996; MacRae & Ulyatt, 1974). This inefficiency is associated with rapid degradation of protein in the rumen meaning that ammonia is generated in excess of that which can be used by the rumen microbiota for protein synthesis (Kolver, Muller, Varga, & Cassidy, 1998). Increasing the efficiency of rumen protein metabolism requires a solution that will decrease production of free ammonia while not inhibiting microbial protein synthesis and feed degradability in the rumen.

The rumen can be considered to be a semi‐continuous flow fermenter in which the microbial consortium degrades the ingested feed, the products of which such as volatile fatty acids (VFA) and protein breakdown products are used to drive microbial growth (Huws et al., 2018). Flow‐through of rumen liquor containing a proportion of the microbiota and nondegraded plant protein (Hart, Onime, Davies, Morphew, & Kingston‐Smith, 2016) from the rumen to the small intestine allows absorption of nutrients into the animal's bloodstream driving animal productivity (Firkins, 1996). It has previously been demonstrated that endogenous plant enzymes responses to rumen conditions could contribute to inefficient protein use during fermentation by the rumen microbiota (Beha, Theodorou, Thomas, & Kingston‐Smith, 2002; Kingston‐Smith, Davies, Edwards, Gay, & Mur, 2012; Kingston‐Smith, Merry, Leemans, Thomas, & Thoeodorou, 2005). Exposure of fresh plant material to the conditions of the rumen can induce stress signaling (Kingston‐Smith et al., 2012) culminating in protein breakdown even in the absence of microbial activity (Zhu et al., 1999; Beha et al., 2002; Kingston‐Smith, Bollard, & Humphreys, 2002). Therefore, modulation of the plant stress response to the rumen conditions could result in decreased plant‐mediated proteolysis and so provide a forage‐based solution to inefficiency of protein use in the rumen (Kingston‐Smith, Marshall, & Moorby, 2013).

According to Shaw (2006), O'Donovan, Kingston‐Smith, and Humphreys (2012) and Humphreys, O’Donovan, Farrell, Gay, and Kingston‐Smith (2014) the rate of plant‐mediated protein breakdown varies in different grass species on exposure to the rumen conditions which imply high temperature (39°C), anoxia and darkness. Shaw (2006) found that the rate of protein breakdown in *L. perenne* and *L. multiflorum* was approximately four times faster when compared to the rate that was observed for *F. arundinacea var. glaucescens* under in vitro rumen‐like conditions*.* This observation is notable as, although they are different species, *Lolium* and *Festuca* can hybridize to generate *Festulolium* hybrids with the potential to enhance advantageous traits in the progeny that are not expressed to the same extent in the parents (heterosis). *Lolium* and *Festuca* species share complementary traits for yield, persistence, and quality so through hybridization there is the potential to produce germplasm which produces large yields of high‐quality forage (Humphreys, Yadav, et al., 2006). Wang and Bughrara (2008) predicted that hybrids between *F. mairei* and either *L. perenne* or *L. multiflorum* would have enhanced stress tolerance mechanisms (particularly enhanced drought tolerance and winter hardiness) enabling survival of hybrids. *Festulolium* grasses have indeed been developed to provide specialist functions and novel alternatives to existing grass cultivars due to their strong resilience against abiotic or biotic stresses as compared with perennial ryegrass (Ghesquière, Humphreys, & Zwierzykowski, 2010; Humphreys, Gasior, Lesniewska‐Bocianowska, Zwierzykowski, & Rapacz, 2006). It has been demonstrated that *F. arundinacea, F. arundinacea var. glaucescens,* or *F. mairei* can contribute genes for drought and heat tolerance to *Festulolium* hybrids (Humphreys et al., 2012). The origins of the tetraploid *F. mairei* (Atlas fescue) from a very hot and dry location in North Africa may be a contributing factor to the hybrid's possession of good cellular mechanism(s) for heat tolerance. Similarly, introgression breeding programs have led to transfer of genes for drought resistance located on *Festuca* chromosome 3, introducing a major source of novel genetic variation for improved resistance to severe drought stress to *Lolium* (Humphreys, Gasior, et al., 2006). Hence, *Festulolium* have been shown to be able to provide specialist functions and novel alternatives to existing grass cultivars due to their strong resilience against abiotic or biotic stresses as compared with perennial ryegrass (Ghesquière et al., 2010). As a result, *Festulolium* varieties have recently gained interest as a source of reliable, productive and nutritive fodder for use in livestock agriculture and for their potential for ecosystem services (Humphreys et al., 2018; MacLeod et al., 2013).

Given that ingested forage undergoes stress‐mediated metabolism on entering the rumen (Zhu et al., 1999; Kingston‐Smith et al., 2012; Kingston‐Smith et al., 2005), and that *Lolium* and *Festuca* species can show significantly different protein degradation it follows that *Festulolium* forage grass hybrids could also have the potential to improve ruminal N use efficiency in a fresh feeding system. We hypothesize that by supplying the rumen with a fresh forage feed containing the slow plant‐mediated proteolysis phenotype, during digestion the supply of protein breakdown products will better match availability of energy (generated by fiber breakdown). In consequence, this will limit the substrate availability for the hyperammonia‐producing bacteria thereby decreasing (but not removing completely) the extent of ammonia production. Previously it has been shown that it is possible to transfer a slow proteolysis trait from *Festuca* to *Festulolium* hybrids (Humphreys et al., 2014). However, those studies were conducted in an in vitro system which lacked rumen microbiota, having been designed to assess only the plant‐mediated proteolysis potential. To fully appreciate the potential of the *Festulolium* hybrids to decrease the environmental impact in a pasture based ruminant system it is important to test these materials under conditions more realistic of those found in the rumen. Therefore, a semi‐continuous flow fermentation system was used to establish whether the presence of a slowed proteolysis phenotype in *Festulolium* hybrids, in comparison with the rate of proteolysis typical in a National List variety of *L. perenne,* could be used to enhance efficient microbial protein synthesis.

## 2 MATERIALS AND METHODS

### 2.1 Plant material

Experimental plants were grown in replicated 3m × 7m field plots, at Gogerddan Campus, IBERS Aberystwyth University (52°25'57.8"N 4°01'04.0"W). Grasses were obtained from monocultures sown in a silt/clay loam soil. For brevity species names and species’ hybrids are described throughout by the following nomenclature: *L. perenne* var AberMagic = Lp (considered as control); *L. perenne* × *F. arundinacea* var. *glaucescens* (*F*_1_) = LpFg; *L. multiflorum* × *F. arundinacea* var. *glaucescens* (*F*_1_) = LmFg; *L. perenne* × *F. mairei* (*F*_1_) = LpFm. Grasses were harvested at the same hour (15:00hr) having a target maturity of reproductive stage R1‐index 3.1 (Moore & Moser, 1995). Selection of these cultivars was based on previous evidence of potential advantages as cultivar or individual genotypes in terms of protein stability and thermotolerance where *F. arundinacea var glaucescens* had been shown previously to have slower rates of endogenous protein degradation than *L. perenne*, and *F. mairei* is one of the most heat tolerant fescues (Humphreys et al., 2014, 2018; MacLeod et al., 2013). All cultivars used were 4x as it was shown by Humphreys et al. (2014) that a balanced *Lolium*: *Festuca* genome was required for expression of the slow proteolysis trait.

### 2.2 Experimental design

Since *Festulolium* hybrids were generated from different paternal plants, high variation was expected within each variety. Thus, for each treatment, a similar amount of grass was collected from each of the three replicate plots and pooled to generate a homogeneous and representative sample of each cross. Grass was harvested daily by cutting at 5 cm above soil level and a dry matter (DM) determination was performed on a subsample to determine the amount of fresh sample required each day to achieve 10gDM/d per fermentation vessel.

### 2.3 Experiment 1: Stress‐induced endogenous proteolysis

Grass harvested from the field plots was cut into 1 cm lengths from which 0.2g was placed in each of 20 Hungate tubes (Scientific Laboratory Supplies Ltd) containing anaerobic phosphate and bicarbonate buffer pH 6.8 (Van Soest, 1967). Tubes were backfilled with anaerobic gas (10% CO_2_, 10% H_2_, 80% N) and sealed with butyl rubber stoppers and incubated at 39°C in the dark in the absence of rumen microbial inoculum for 24 hr as described by Kingston‐Smith, Davies, Rees Stevens, and Mur (2013). Four tubes per cultivar were sampled at 0, 2, 4, 6, 24 hr of incubation. Before sampling, tubes were gentle inverted and plant material was recovered by filtration and washed with 50 ml of deionized water per sample. Samples were then placed into 1.5ml microfuge tubes, frozen in liquid N and stored at −80°C until further analyses. Soluble protein was extracted from frozen samples by grinding grass leaf blades to a fine powder in liquid N. This was homogenized in 1 ml of extraction buffer [0.1 M HEPES buffer, pH 7.5 containing 2 mM EDTA, 1 mM dithiothreitol, 0.1% (v/v) Triton X‐100, 0.5% (v/v) protease inhibitor cocktail] (Sigma UK Ltd, Gillingham, UK) at the ratio of 10 ml/g FW. Extracts were centrifuged at 10,000×g for 10 min at 4°C, after which the supernatant was transferred into clean tubes. Then, protein content of extracts was determined according to Bradford (1976).

### 2.4 Experiment 2: Rumen simulation technique (Rusitec)

To understand if the differences in plant‐mediated proteolysis observed in the in vitro system (as described above) were significant in the presence of a rumen microbial inoculum, a continuous culture system (Rusitec) was used to explore the forage degradation parameters across the four grass treatments (Czerkawski & Breckenridge, 1977). Experimentation was conducted under the authority of licenses under the U.K. Animal Scientific Procedures Act, 1986 and managed according to the protocols approved by the Aberystwyth University Animal Welfare and Ethics Review Board. Grasses harvested from the field plots were cut at 5 cm above the soil level, and stored at 4°C for 12 hr until the daily DM content was determined for each grass. Harvested grasses were chopped into 1 cm leaf blades and the equivalent to 10g DM of grass was placed into nylon bags (50 µm pore size; 10 cm × 20 cm) to be incubated in each fermenter for 48 hr. The experimentation consisted of a single incubation period including 16 vessels as experimental units. Thus, each treatment had 4 replicates which were randomly allocated to the vessels and inoculated with rumen fluid from 4 cows. Each vessel was treated as an experimental unit and consisted of airtight 900 ml vessels immersed in a water bath maintained at 39°C and provided with permanent vertical agitation. Rumen fluids and solids (2.5L/cow and 200g/cow respectively) were collected before the morning feeding from four rumen‐cannulated cows. These cows had been fed on 80% ryegrass silage and 20% concentrate on DM basis. The rumen contents were strained through a double layer of muslin into heat‐retaining flasks allowing the contents to be maintained at 39°C and transferred within 1 hr to the in vitro system. Rumen fluid from each cow was used as independent inocula and distributed in 4 vessels each of which was incubated with one of the 4 grasses (*n* = 4). Artificial saliva (pH 8.4) was freshly prepared every day and continuously infused to each vessel at a rate of 614 ml/day (dilution rate 0.0365/hr) using a multi‐channel peristaltic pump.

### 2.5 In vitro system adaptation and sampling

Rusitec incubation was performed as previously described (Belanche, Jones, Parveen, & Newbold, 2016). Briefly, on day 1 of the experiment, each vessel was inoculated with 500 ml of rumen fluid, 400 ml of artificial saliva reduced with resazurin at 0.1% per liter (McDougall, 1948), one nylon bag containing the forage of interest and another bag containing 10g FM of rumen solids in order to supply solid‐associated bacteria. Vessels were bubbled with CO_2_ during feeding to maintain anaerobic conditions. The displaced effluent from each of the fermentation vessels was collected in effluent bottles while the fermentation gasses were collected into gas collection bags. Vessels were opened and each bag containing solid rumen digesta was replaced by a new bag containing forage. On subsequent days, the nylon bag that had been in the vessel for 48 hr was rinsed with 50 ml of artificial saliva and this saliva was returned back to the fermenter together with a new bag containing forage.

The Rusitec system was operated for 14 days. The first nine days were used for microbial adaptation and to stabilize fermentation (Belanche, Lee, Moorby, & Newbold, 2013) during which the amount of overflow, pH, and gas volume from each vessel was measured to ensure that conditions in all the vessels were similar. In the incubation period between days 1 and 11, 10 ml of 20% H_2_SO_4_ was placed into each of the 16 overflow flasks daily to stop fermentation.

On Day 9, 3 mg of ^15^N was added to each fermenter to label the ammonia pool, equivalent of 13 mg of 99% labeled (^15^NH_4_)_2_SO_4_. From Day 9 onwards, the ^14^N‐ammonium sulfate used in the preparation of artificial saliva was replaced with by ^15^N‐ammonium sulfate (3.7 mg of ^15^N/L per liter; equal to 17 mg/L of 99% labeled (^15^NH_4_)_2_SO_4_ to label the ammonia‐N pool to measure microbial protein synthesis (MPS). On days 9, 10, and 11, the vessel pH as recorded and along with the amount of gas produced, moreover methane concentration was measured using a gas analyser (SR8610C Gas Chromatograph). On these 3 days, bags which were incubated for 48 h were rinsed 5 s with tap water to determine feed disappearance. An aliquot of 1 ml of vessel content was daily collected before feeding and placed in 4% formaldehyde for use in protozoal quantification and classification by optical microscopy (Dehority, 1993).

On days 12 and 13, sampling for microbial protein synthesis was conducted (Carro & Miller, 1999). The H_2_SO_4_ in the overflow bottles was replaced with 10ml of saturated HgCl_2_ diluted at 1:5 and all overflow bottles kept on an ice‐water bath to prevent microbial growth. Fermentation gasses were collected into hermetic bags to determine gas production and methane concentrations. Overflow was collected from early (0–8 hr) and late fermentation (8–24 hr) after forage supply. Overflow from each vessel was collected during these 2 days, pooled, and kept cold. One sample was taken (1 ml mixed with 0.25 ml of 25% trichloroacetate) for ammonia determination while other sample (4ml mixed with 1 ml of ortho‐phosphoric acid 20% and 10 mM of 2‐ethylbutyric acid as an internal standard) to quantify volatile fatty acids (VFA).

The bag containing the 48 hr plant residue from the two days was combined, and then 1/3 was combined with the early fermentation overflow and 2/3 with the late fermentation overflow in order to reconstitute the total digesta overflow. One aliquoted (100ml) was freeze‐dried to determine DM and ^15^N enrichment in non‐ammonia‐N (NAN) whereas the second aliquot (50ml) was used to isolate the liquid associated bacteria (LAB) pellet.

For NAN determination, freeze‐dried digesta (1.5g DM) was mixed with NaOH (1M) until pH was above 10 and left at room temperature for 30 min for ammonia volatilization (Firkins, Weiss, & Piwonka, 1992). The resulting residue was freeze‐dried again for N and ^15^N determinations. For LAB isolation, the digesta overflow was centrifuged at 500 g for 10 min to remove plant particles and protozoa. The supernatant was centrifuged at 10,000 *g* for 25 min to sediment the LAB community whereas the supernatant was kept frozen for ammonia ^15^N analysis. This centrifugation was repeated to clean the LAB with 0.9% NaCl prior freeze‐drying.

For the ammonia ^15^N analysis, ammonia‐N was extracted by the diffusion method (Brooks, Stark, McInteer, & Preston, 1989) in duplicate from the supernatant obtained. Supernatant (45 ml) was mixed with 5 ml of 65% TCA and incubated for 24 hr at 4°C to precipitate the proteins. This was followed by centrifugation at 10,000 *g* for 25 min at 4°C and the resultant supernatant (35 ml) was transferred to a new tube and 5ml of 15M NaOH was added to increase pH above 10. Ammonia evaporated over 7 dayghn ncnnnnnncn at room temperature and was capture onto 5 mm glass microfiber disks (Whatman GF/D) impregnated with 20 ml of 2.5 M KHSO_4_. Disks were dried in a desiccator (with the atmosphere saturated in H_2_SO_4_) for 24 hr before being analyzed for ^15^N enrichment.

For SAB determination, 48 hr bag residues were washed twice with 50ml of artificial saliva to remove loosely associated bacteria and the residue was then frozen. The residue was defrosted and suspended with 100 ml of saline solution (0.9%) containing carboxy‐methyl‐cellulose (0.1%) and incubated for 30 min at 39°C. The samples were detached using a Stomacher (Seward) for 2 min at low speed and incubated at 4°C for 4 hr. These were then filtered through muslin and centrifuged at 500 *g* for 10 min to remove plant particles. The supernatant was centrifuged at 10,000 *g* for 25 min to sediment SAB. This centrifugation was repeated again to clean the SAB community. The SAB community was freeze‐dried and used for ^15^N analysis.

### 2.6 Analytical procedures

The DM content of samples was determined by drying in an oven at 105°C for 24 hr. Ash concentration was determined by heating at 550°C for 6 hr in a muffle furnace and the organic matter (OM) concentration was calculated by mass difference. The concentration of water‐soluble carbohydrate (WSC) in the plant material was determined by spectrometry using anthrone in sulfuric acid on a Technicon AutoAnalyzer (Technicon Corporation) (Thomas, 1977). The concentration of non‐ammonia‐*N* (NAN) was determined by a micro‐Kjeldahl technique using Kjeltec equipment (Perstorp Analytical Ltd) after ammonia evaporation by adding NaOH (pH > 10). Concentration of neutral detergent fiber (NDF) was determined using heat stable amylase and sodium sulfite as described by Van Soest, Robertson, and Lewis (1991). Acid detergent fiber (ADF) concentration was analyzed using the Tecator Fibertec System (Tecator Ltd, Thornbury, Bristol). Both fibers were expressed inclusive of residual ash. Concentration of VFAs in the fermentation liquor was determined by Gas Chromatography (Richardson, Calder, Stewart, & Smith, 1989). Ammonia‐N concentration was determined by a colorimetric method (Weatherburn, 1967). The volume of gas produced was measured with a drum‐type gas meter (Series TG1, Ritter Apparatebau GmbH, Bochum, Germany) while the methane concentration was determined by Gas Chromatography (Carro & Miller, 1999) and expressed at normal conditions (1 atm and 20°C). Abundance of ^15^N was determined using an N analyser connected to a mass spectrophotometer (ANCA/SL 20/20, PDZ Europa Ltd, Crewe).

### 2.7 Statistical analyses

Microbial N contribution to overflow was calculated based on the relationship between the ^15^N enrichment in the digesta (NAN) and in the LAB or SAB communities (Microbial N: NAN = Digesta NAN ^15^N enrichment: bacterial pellet ^15^N enrichment). The ability of LAB and SAB to incorporate ammonia was calculated as the ratio between their ^15^N enrichments (bacterial ^15^N enrichment: ammonia ^15^N enrichment). The differences in the forages were statistically determined by general ANOVA using Genstats 16th Edition, Release 16.1 (VSN International Ltd, Hemel Hempstead, UK) as follows:

$$Y_{\mathrm{ijk}}=\mu +G_{i}+A_{j}+e_{\mathrm{ijk}}$$

where *Y_ijk_* is the dependent, continuous variable, *µ* is the overall mean; *G_i_* is the fixed effect of the grass cultivars (*i* = Lp versus LpFg versus LmFg versus LpFm), *A_j_* is the random effect of the animal inoculum (*j* = 1 to 4) and *e_ijk_* is the residual error. When significant effects were detected, treatment means were compared by Fisher's protected LSD‐test. Findings with *p* < .05 were regarded statistically significant while *p* < .1 was considered as a tendency to differences.

## 3 RESULTS

### 3.1 Experiment 1: Stress‐induced endogenous proteolysis

**Figure 1:**
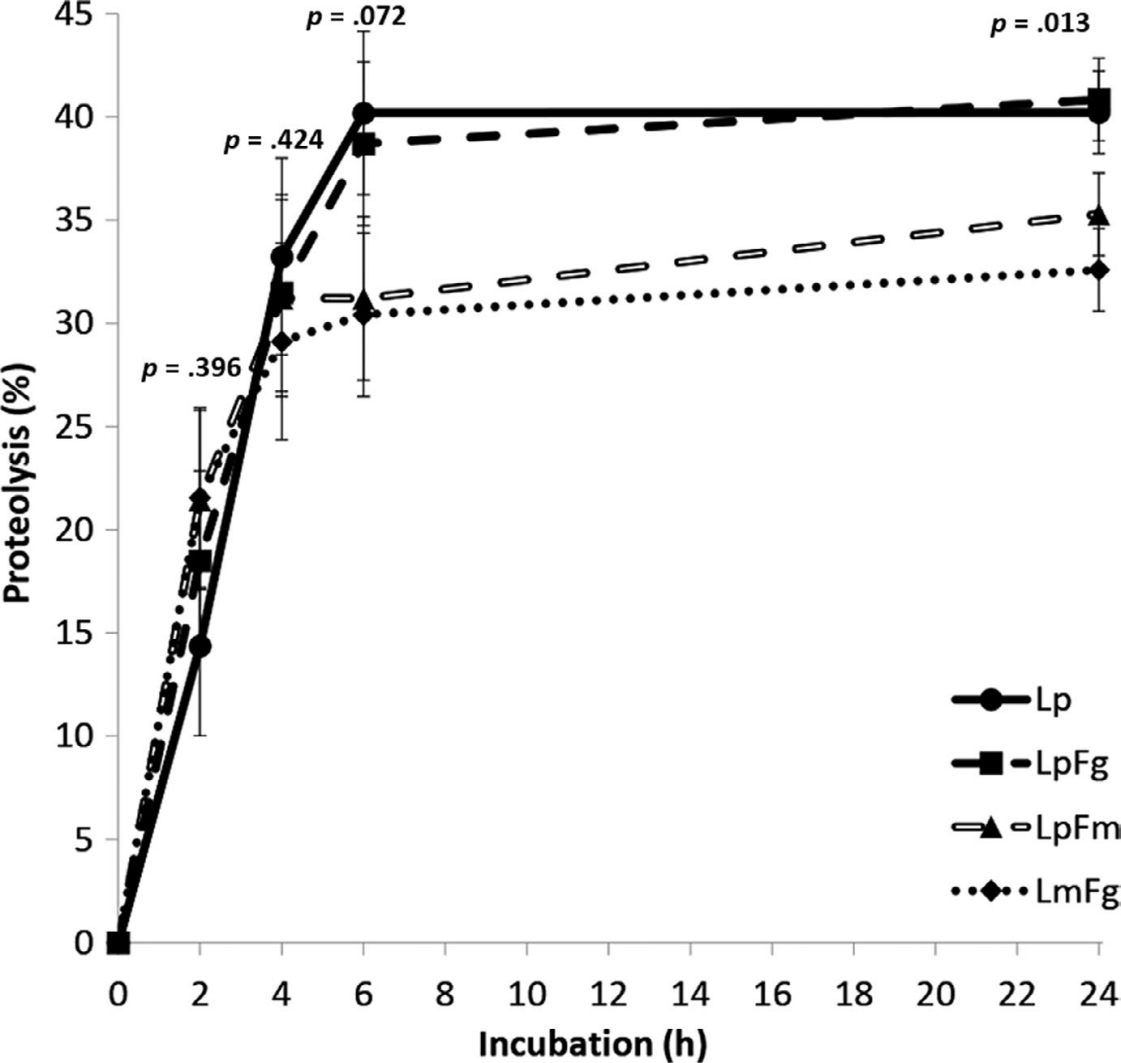
Rate of plant‐mediated auto‐proteolysis of different forage cultivars at different incubation times in anaerobic buffer at 39ºC. Treatments: Lp, *Lolium perenne*; LpFg, *Lolium perenne* × *Festuca arundinacea var. glaucescens*; LpFm, *Lolium perenne* × *Festuca mairei*; LmFg, *Lolium multiflorum* × *Festuca arundinacea var. glaucescens*

Some differences were noted in terms of feed composition across treatments (Table 1). Hybrid FmFg had the greatest concentrations of N and ADL but the lowest of NDF, while the opposite was true for LpFg and LpFm hybrids. In addition, Lp had intermediate concentrations of N and ADL in comparison with festulolium hybrids. The Grass leaf blades were incubated for between 0 and 24 hr in anaerobic buffer at 39°C which exposed them to a combination of heat stress and anoxia as would be experienced in the rumen. The effect of exposure to the stress conditions on soluble protein content of the leaf blades was determined. Results showed that as incubation time progressed, protein content decreased in all treatments suggesting stress‐induced autolysis of leaf protein was occurring. Substantial protein loss occurred between 0 and 6 hr of incubation with minimal losses observed between 6 and 24 hr across treatments (Figure 1). Although no significant differences between forage cultivars were detected during the first 4 hr of incubation, Lp and Lp × Fg showed higher rates of plant‐mediated auto‐proteolysis during the 4 to 6 hr time interval after initiating the incubation suggesting a potential source of slow‐release protein. As a result, Lp and Lp × Fg showed higher levels of protein degradation at 6 hr (*p* = .072) and 24 hr of incubation (*p* = .013) in comparison with Lp × Fm and Lm × Fg hybrids.

**Table 1:** Chemical composition (in % DM) of the forage grass cultivars used for plant‐mediated proteolysis and rumen simulation technique

Grass cultivars^1^	Lp	LpFg	LpFm	LmFg	s.e.d^2^	*p*‐value
OM	92.2	92.1	94.0	91.9	0.216	.260
N	1.95^b^	1.65^c^	1.71^c^	2.11^a^	0.057	<.001
WSC	24.1	27.8	28.2	28.2	0.369	.096
NDF	40.1^a^	37.8^a^	40.0^a^	31.0^b^	1.600	.024
ADF	20.6	19.8	19.2	17.4	1.828	.850
ADL	2.22^ab^	1.57^b^	1.89^b^	3.97^a^	0.511	.049

Abbreviations: N, nitrogen; WSC, water‐soluble carbohydrate; NDF, neutral detergent fiber; ADF, acid detergent fiber; ADL, acid detergent lignin.

^1^ Lp, *Lolium perenne*; LpFg, *Lolium perenne* × *Festuca arundinacea var. glaucescens*; LpFm, *Lolium perenne* × *Festuca mairei*; LmFg, *Lolium multiflorum* × *Festuca arundinacea var. glaucescens.*

^2^ Standard error of the difference (*n* = 4). Different letters within the same row indicate significant differences between means (*p* < .05). Means are indicated for OM, organic matter;

### 3.2 Experiment 2: Forage digestibility and gas production in Rusitec

When the four forages (Lp, and three *Festulolium* hybrids) were used as fermentation substrates, in general, there were no genotypic effects on feed digestibility (Table 2). There were no significant differences between the forages in terms of the disappearance of organic matter (OM), total N (N), water‐soluble carbohydrates (WSC), dry matter (DM), neutral detergent fiber (NDF), or acid detergent fiber (ADF; Table 2). Nonsignificant differences in total gas and methane production between forage substrates were noted in this experiment indicating that control of protein metabolism was independent of fermentation kinetics.

**Table 2:** Digestibility and gas production of *Lolium perenne* and *Festulolium* hybrids

Grass cultivars^1^	Lp	LpFg	LpFm	LmFg	s.e.d^3^	*p*‐value
Disappearance^2^ (%)						
Dry matter	67.3	71.4	66.8	63.7	6.04	.660
Organic matter	66.3	71.3	66.0	63.9	6.20	.687
Nitrogen	72.2	71.6	72.6	63.1	7.29	.567
Water‐soluble carbohydrates	97.6	98.4	96.8	96.8	1.26	.567
Neutral detergent fiber	48.1	52.7	33.9	44.0	8.68	.233
Acid detergent fiber	42.0	47.4	31.8	36.0	7.69	.265
Gas emissions						
Total gas (l/d)	1.89	1.69	2.04	1.85	0.224	.531
Methane (%)	4.4	3.94	4.21	4.18	0.727	.936
Methane (ml/d)	83.7	67.4	85.7	81.5	20.01	.796

^1^ Lp, *Lolium perenne*; LpFg, *Lolium perenne* × *Festuca arundinacea var. glaucescens*; LpFm, *Lolium perenne* × *Festuca mairei*; LmFg, *Lolium multiflorum* × *Festuca arundinacea var. glaucescens.*

^2^ Mean values of samples recovered 48 hr after forage administration.

^3^ Standard error of the difference (*n* = 4).

### 3.3 Fermentation and microbial protein synthesis in Rusitec

The fermentation end‐products VFA (volatile fatty acids) were measured during the early (0–8 hr) and late (8–24 hr) stages of fermentation. Total VFA overflow was not significantly different between the cultivars at all stages of fermentation (Table 3), although LmFg and LpFm tended to produced more VFA than Lp or LpFg mostly during the early fermentation (*p* = .10). There was no detectable difference in the production of acetate and propionate whereas butyrate production differed between cultivars in both early and late fermentation. In particular LmFg promoted a highest production of butyrate (followed by LpFm) during the early (*p* = .016) and late fermentation (*p* = .044) resulting in a higher overall butyrate production (*p* = .007) in comparison with the other grass cultivars. Production of branched‐chain volatile fatty acids (isobutyrate, isovalerate and 2‐methyl butyrate; BCVFA) also differed between treatment indicating a higher production for LmFg (followed by FpFm) during the early (*p* = .032), late (*p* = .078) and overall BCVFA production (*p* = .035).

**Table 3:** Fermentation end products of four forage cultivars in rumen simulation technique measured during the early (0–8 hr) and late (8–24 hr) periods of the fermentations

Grass cultivars^1^	Lp	LpFg	LpFm	LmFg	s.e.d^2^	*p*‐value
Total VFA						
Early (mmol/hr)	1.13	1.04	1.33	1.58	0.202	.103
Late (mmol/hr)	0.91	0.76	0.98	1.02	0.141	.332
Total (mmol/day)	23.6	20.5	26.2	28.9	3.32	.139
Acetate						
Early (mmol/hr)	0.55	0.47	0.61	0.71	0.115	.253
Late (mmol/hr)	0.44	0.34	0.45	0.46	0.08	.452
Total (mmol/day)	11.3	9.2	12.1	13	1.941	.299
Propionate						
Early (mmol/hr)	0.28	0.23	0.33	0.36	0.055	.170
Late (mmol/hr)	0.23	0.16	0.23	0.21	0.042	.381
Total (mmol/day)	5.9	4.39	6.24	6.28	0.974	.239
Butyrate						
Early (mmol/hr)	0.14^b^	0.15^b^	0.18^ab^	0.24^a^	0.027	.016
Late (mmol/hr)	0.11^b^	0.11^b^	0.13^ab^	0.16^a^	0.016	.044
Total (mmol/day)	2.81^b^	3.03^b^	3.56^b^	4.45^a^	0.372	.007
BCVFA						
Early (mmol/hr)	0.076^b^	0.072^b^	0.083^b^	0.110^a^	0.0113	.032
Late (mmol/hr)	0.062	0.052	0.065	0.073	0.0067	.078
Total (mmol/day)	1.601^b^	1.399^b^	1.710^ab^	2.045^a^	0.1811	.035

Abbreviations: BCVFA, branched‐chain volatile fatty acids; VFA, volatile fatty acids.

^1^ Lp, *Lolium perenne*; LpFg, *Lolium perenne* × *Festuca arundinacea var. glaucescens*; LpFm, *Lolium perenne* × *Festuca mairei*; LmFg, *Lolium multiflorum* × *Festuca arundinacea var. glaucescens.*

^2^ Standard error of the difference (*n* = 4). Different letters within the same row indicate significant differences between means (*p* < .05)

In terms of N flows (Table 4), there was a general higher outflows of total N, NAN, ammonia‐N, and microbial N during the early in comparison with the late fermentation stage. Moreover, during the early fermentation Lp promoted a higher total N overflow than *Festulolium* hybrids (*p* = .010) while LpFm and LmFg showed the greatest N flows during the late fermentation (*p* = .022). As a result, similar daily overflow of total N was found for Lp, LpFm, and LmFg but lower for LpFg treatment (*p* = .019). In contrast, the amount of NAN produced was not significantly different between cultivars. All *Festulolium* hybrids tended to produce lower ammonia‐N overflow than Lp during the early fermentation (*p* = .065) while during the late fermentation the greatest and lowest values were observed in LmFg and LpFg hybrids respectively (*p* = .018). As a result, similar daily ammonia‐N flow was observed across treatments except for LpFg which showed the lowest values (*p* = .034).

**Table 4:** Nitrogen flows during fermentation of forage cultivars in rumen simulation technique measured during the early (0–8 hr) and late (8–24 hr) periods of the fermentations

Grass cultivars^1^	Lp	LpFg	LpFm	LmFg	s.e.d^2^	*p*‐value
Total *N*						
Early (mg/hr)	8.56^a^	6.96^b^	7.02^b^	7.44^b^	0.398	.010
Late (mg/hr)	6.24^ab^	5.53^b^	6.96^a^	6.90^a^	0.410	.022
Total (mg/day)	168^a^	144^b^	168^a^	170^a^	7.300	.019
Non‐ammonia‐*N*						
Early (mg/hr)	6.12	4.97	5.13	5.44	0.374	.056
Late (mg/hr)	4.39	4.23	5.30	4.69	0.442	.146
Total (mg/day)	119	107	126	119	7.820	.198
Ammonia‐*N*						
Early (mg/hr)	2.44	1.98	1.90	1.99	0.188	.065
Late (mg/hr)	1.85^ab^	1.30^c^	1.66^bc^	2.20^a^	0.222	.018
Total (mg/day)	49.1^a^	36.7^b^	41.7^ab^	51.2^a^	4.460	.034
Microbial *N* with LAB						
Early (mg/hr)	4.18	3.48	3.88	4.01	0.44	.464
Late (mg/hr)	2.29^c^	2.21^c^	2.86^b^	3.36^a^	0.144	<.001
Total (mg/day)	70.2^bc^	63.1^c^	76.9^ab^	85.8^a^	5.010	.008
Microbial *N* with SAB						
Early (mg/hr)	3.98	3.32	3.88	3.93	0.446	.458
Late (mg/hr)	2.31^b^	2.45^ab^	3.11^a^	3.13^a^	0.307	.047
Total (mg/day)	68.8	65.8	80.9	81.6	7.650	.150
Microbial *N* from ammonia						
LAB (%)	48.8	48.1	49.0	47.8	2.85	.966
SAB (%)	50.2	46.3	46.9	50.6	6.283	.864
Efficiency of microbial synthesis						
g LAB‐N/kg OM disappeared	10.7^bc^	8.90^c^	11.7^ab^	13.5^a^	1.020	.009
g LAB‐N/kg N disappeared	503^b^	537^b^	503^b^	819^a^	74.70	.006
g SAB‐N/kg OM disappeared	9.29	9.29	12.1	13.2	1.907	.242
g SAB‐N/kg N disappeared	491	561	523	811	141.7	.169

^1^ Lp, *Lolium perenne*; LpFg, *Lolium perenne* × *Festuca arundinacea var. glaucescens*; LpFm, *Lolium perenne* × *Festuca mairei*; LmFg, *Lolium multiflorum* × *Festuca arundinacea var. glaucescens.*

^2^ Standard error of the difference (*n* = 4). Different letters within the same row indicate significant differences between means (*p* < .05).

Given the variability in the chemical composition across the microbial groups in the rumen, the LAB and SAB microbial communities were used to estimate the microbial protein synthesis. Both microbial communities showed similar estimates indicating no differences in the microbial N overflow across treatments during the early fermentation. However, during the late fermentation a higher microbial protein overflow was noted in vessels containing LmFg (followed by LpFm) than for Lp and LpFg when considering the LAB (*p* < .001) and SAB (*p* = .047), respectively. As a result, LmFg promoted the greatest overall microbial N overflow across treatments with respect to LAB (*p* = .008), but it was less evident when considering SAB community (*p* = .15). There was no genotypic effect detected on the overall proportions of LAB‐N and SAB‐N derived from ammonia‐N indicating a constant ammonia‐N uptake for LAB and SAB (average 48.5%) across treatments. In terms of efficiency of microbial protein synthesis, LmFg (followed by LpFm) promoted the greatest efficiency in terms of microbial N flow per unit of OM (*p* = .009) and N disappearance (*p* = .006) when using LAB as reference. On the contrary, LpFg promoted the lowest efficiency values across treatments. LmFg also showed the greatest microbial N efficiency values across treatments when the SAB community was used as reference; however, those differences did not reach statistical signification (*p* < .25).

## 4 DISCUSSION

Selective grass breeding has been conducted on LmFg BC_2_ introgression lines on the basis of their heat and drought tolerance toward producing a more stress‐tolerant and current UK National Listed variety; AberLink (Humphreys, Harper, Armstead, & Humphreys, 2005a; APHA, 2017). Hence, they may be viewed as possible alternatives to the use of seed mixtures, or for a specialist use such as in drought‐prone regions or in preventing flooding due to deep rooting, for example, MacLeod et al., (2007); MacLeod et al. (2013); Humphreys et al. (2018). Theoretically, the hybridization of nutritionally desirable *Lolium* with stress‐tolerant *Festuca* species would not only help protect the crop in a harsh field environment but could affect the cellular responses on exposure to the adverse conditions of the rumen. Shaw (2006) suggested that the heat tolerance derived from the *F. arundinacea var. glaucescens* genes in *L. multiflorum* provide some co‐adaptation to rumen stress conditions.

In this study, we tested the potential for *Festulolium* hybrids to deliver improved protein protection to improve ruminant N use efficiency as compared with a UK National Listed forage ryegrass (*Lolium*). *Lolium* grasses are widely considered to be the forage of choice for ruminants because of their desirable nutritional qualities, particularly in terms of their high growth rate, protein content, and high digestibility (Kingston‐Smith, Marshall, et al., 2013). The determination of the protein content in *Festulolium* hybrids and Lp as control (Table 1) effectively demonstrated that the Lp sward contained higher N content (+18% and + 14%) than LpFg and LpFm hybrids, respectively, but slightly lower (−7%) than LmFg. These findings are consistent with previous work that found *Lolium* parental genotypes to have consistently higher initial protein content than *Festuca* genotypes and progeny (Humphreys et al., 2014; Shaw, 2006). While a good supply of feed protein breakdown is necessary to provide the substrates for microbial growth in the rumen, an excess of feed protein has negative consequences for the animal and environment. Under conditions of excessive breakdown of plant proteins in the rumen, the availability of ammonia and free amino acids in the rumen can exceed the microbial uptake capacity required for growth without further increments in the microbial protein synthesis (Bach, Calsamiglia, & Stern, 2005). This effect is particularly evident in grazing situations when high proteolysis rates during the early fermentation are combined with a situation of energy‐limitation due to the slow degradation of complex carbohydrates. On the contrary, the availability of energy during the late fermentation (mostly from fiber degradation) is often coupled with a limited N availability due to the lack of slow‐release proteins. This asynchronous nutrient availability can limit the microbial growth (Sinclair, Garnsworthy, Newbold, & Buttery, 1995), the excess of rumen N availability during the early fermentation has been associated to increased urinary N excretion (MacRae & Ulyatt, 1974) while the excess of rumen energy availability often leads to energy spilling reactions which waste energy in futile cycles of protons thought the cell membrane or by cycles of synthesis and catabolism of glycogen (Hackmann & Firkins, 2015). Here, exposure of the Lp and *Festulolium* hybrid grasses to conditions of mild heat stress (39°C) and lack of oxygen in the dark (as would be experienced in the rumen) resulted in a net loss of protein indicative of stress‐induced autolysis of leaf protein as described previously (Beha et al., 2002; Demirevska‐Kepova, Holzer, Simova‐Stoilova, & Feller, 2005; Kingston‐Smith, Bollard, Armstead, Thomas, & Thoeodorou, 2003). Furthermore, the *Festulolium* hybrids LpFm and LmFg were confirmed as having a slower (−23%) endogenous protein autolysis than the Lp control during the early fermentation (4 to 6 hr interval, Figure 1), thus they could be considered as a source of slow‐release N.

To understand the extent to which the observed differences in grass protein degradation phenotype would translate to improved ruminal N use efficiency, the rumen fermentation pattern and microbial protein synthesis were assessed using the Rusitec system. Fermentation parameters were remarkably similar for the four grasses. Similarities in methane and total gas production (Table 2) could be attributed to the similar chemical composition profile and nonexistent differences in the digestibility value of Lp and hybrids. Furthermore, all four cultivars produced similar amounts of total VFA, presumably due to them containing similar amounts of potentially fermentable carbohydrates and digestible NDF (Table 1). This observation is in line with the lack of differences in the OM disappearance and seems to indicate a similar fermentable energy supply to rumen microbes across all grass cultivars. However, there was a plant genotypic effect on butyrate production over the entire fermentation process such that LmFg promoted higher (+59%) butyrate production than Lp. Considering that butyrate is a fermentation product which is positively associated with the easily fermentable carbohydrates such as WSC, hexoses, and glutarate (Noziere, Glasser, & Sauvant, 2011), it seems to indicate a sufficient energy availability in the LmFg hybrid. This maintained energy availability along with the delayed N breakdown observed in festulolium hybrids could facilitate the microbial protein synthesis and fiber degradation during the late fermentation since fibrolytic microbes are sensitive to an N shortage (Belanche et al., 2012). These novel results require however further confirmation.

Assessment of plant‐mediated proteolysis in an in vitro system showed that the rate of proteolysis in Lp was faster than in LmFg and LpFm but similar to that seen in LpFg (Figure 1). A relationship between leaf protein content and autolytic rate has been described previously for non‐nodulating white clover mutants where leaf protein content could be altered via root N supply (Kingston‐Smith, Bollard, & Minchin, 2006). Rapid and extensive proteolysis in the rumen will result in an excess of ruminal ammonia production which cannot be absorbed by the animal and is excreted onto the land with consequential issues of N pollution (Kingston‐Smith, Marshall, et al., 2013; Kolver et al., 1998). Thus, if forages can be developed to contain a low proteolysis trait then production of excessive levels of ruminal ammonia (i.e., still sufficient to support rumen microbial growth) will be minimized as will N pollution of farmland. However, the relationship between rate of proteolysis and initial protein did not hold true for LpFg which showed similar rates of proteolysis to Lp but significantly lower initial protein content (Figure 1) suggesting a more elegant mechanism of proteolysis control may operate. There was no evidence of a relationship between rate of proteolysis and initial N content, possibly because of the inclusion of non‐protein N in this measurement.

When placed in Rusitec, the rumen digestibility values for N disappearance across grass cultivars ranged from 63% to 73%, these values being similar to those reported in previous Rusitec studies when using the same Lp cultivar as either fresh plant material (Belanche, Jones, et al., 2016; Belanche, Newbold, Lin, Stevens, & Kingston‐Smith, 2017) or preserved as hay (Belanche et al., 2013). Importantly though, this study revealed that the pattern of N utilization differed between Lp and the *Festulolium* hybrids LpFm and LmFg which expressed slowed proteolysis compared with Lp. On average all three *Festulolium* hybrids tended to promote lower overflows of total‐N (average −17%), non‐ammonia‐*N* (−15%), and ammonia‐*N* (−20%) during the early fermentation in comparison with vessels incubated with Lp. This lower ammonia‐N overflow seems to indicate a lower (or delayed) proteolysis rate in the *Festulolium* hybrids during the early fermentation. This delayed protein breakdown in *Festulolium* hybrids had positive effects on the microbial protein synthesis for LpFm and LmFg (Table 5) indicating a sufficient availability of N and energy for the microbes during the late fermentation (Bach et al., 2005). This is in line with previous observations that autolytic rate in progeny was under genetic control (Humphreys et al., 2014) such that “slow‐degrader” qualities from respective *Festuca* parental genotypes (that are generally slow protein degraders) can be inherited and expressed in hybrids (Shaw, 2006).

Notably, there was a benefit from the observed delay in protein breakdown (mainly LmFg) as it led to increased microbial protein overflows during the late fermentation. For instance fermentation of LmFg promoted a higher microbial protein synthesis (+45% using and + 35% for LAB and SAB estimates) during the late fermentation than was present in those incubations with Lp as the substrate. This increment was present but less obvious for LpFm hybrids (+25% and + 35% for LAB and SAB estimates). As a result of this, LmFg and LpFm hybrids (which both expressed slower proteolysis compared with Lp or LpFg) showed an increased total microbial N flow (+ 22% and + 9.5%, respectively) based on LAB estimates suggesting an optimized microbial N synthesis for these hybrids in comparison with Lp. These effects were less obvious when SAB was used as reference to estimate microbial N flows given the intrinsic methodological variability observed during the process of SAB isolation (Belanche et al., 2011). It has been reported that synchronization of ruminal degradation of carbohydrates and N improved the capture of ruminal N by the microbes in lactating cows (Kolver et al., 1998). Thus, our results support the hypothesis that slower rates of stress‐induced proteolysis limited the availability of protein breakdown products and so were better matched to the energy available in the rumen microbes (Bach *et al.*, 2005) resulting in more efficient N assimilation within fermenters administered with some *Festulolium* hybrids than those supplemented with Lp.

On average all three *Festulolium* hybrids tended to have higher WSC (+16.6%) than Lp. Based on the rumen synchronization theory (Hall & Huntington, 2008), high WSC concentrations would be an advantage since soluble carbohydrates in forage provide a source of rapidly available energy for facilitation of microbial protein synthesis during the early rumen fermentation that translates to improved ruminant production in terms of milk and animal growth (Lee et al., 2001; Miller et al., 2001). However, production responses to ryegrass WSC concentrations have been very variable (Parsons et al., 2011). Previous studies on fresh grass‐based diets have suggested an existence of a curvilinear relationship between the dietary ratio of WSC to N and the proportional excretion of dietary N in urine (Parsons et al., 2011) which suggests that a high diet WSC: N ratio would increase N‐use efficiency despite milk production remaining constant (Kingston‐Smith, Marshall, et al., 2013). Similarly, a negative correlation has been reported between the ammonia concentration and the efficiency of N utilization (*R* ^2^ = 0.78) in continuous fermenters (Bach *et al.*, 2005). In our study, LmFg showed the greatest efficiency of microbial protein synthesis in comparison with Lp per unit of OM disappeared (+26.6%) and per unit of N disappeared (+64%) suggesting a higher efficiency of energy and N utilization. LpFg hybrids also showed a slightly higher efficiency of energy utilization for microbial growth (+9.4%) than Lp (as microbial N per unit of OM disappeared) but not in terms of N utilization (as microbial N per unit of N disappeared).

**Figure 2:**
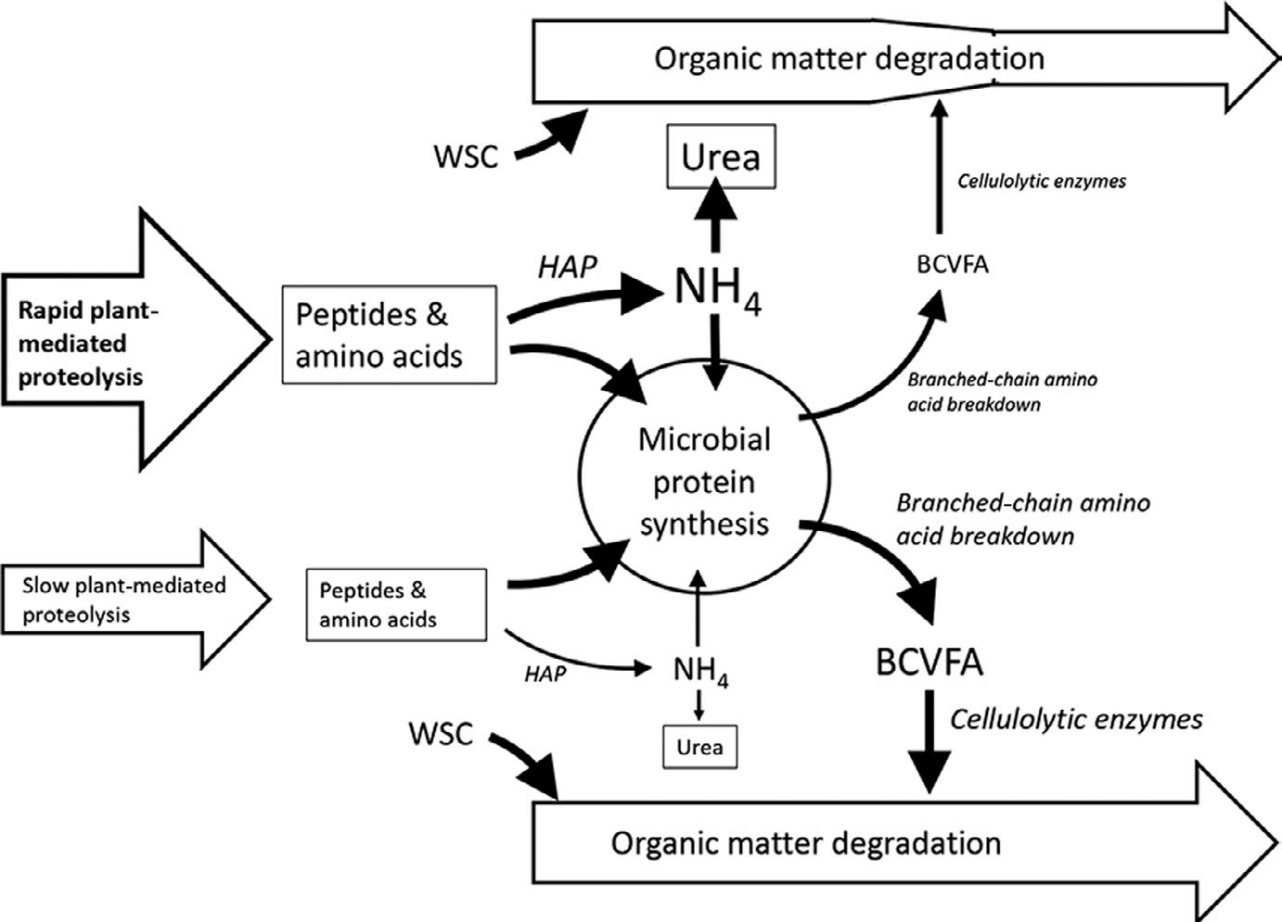
Schematic representation of the proposed mechanism for the central role of differential routes of amino acid catabolism in determining efficiency of ruminal N use. Font and changes in arrow thickness are indicative of changes in relative activities/ abundances. BCVFA: branched‐chain volatile fatty acids; HAP: hyperammonia‐producing bacteria; WS: water‐soluble carbohydrate

Alternatively, the improved protein use efficiency may be due to the alteration in the relative proportions of BCVFA produced in fermentations supplied with plants with fast or slower rates of plant‐mediated proteolysis. These compounds are derived from protein breakdown and are essential for growth of rumen cellulolytic bacteria (Bryant, 1973; Cummins & Papas, 1985). Whole animal trials have shown that supplementation with BCVFA causes increased population of cellulolytic bacteria (*Ruminococcus albus, R. flavefaciens, Butryvibrio fibrisolvens, Fibrobacter succinogenes, Prevotella ruminicola, Ruminiobacter amylophilus*; Wang et al., 2019). Also, enhanced production of BCVFA has been observed in RUSITEC supplied with high levels of free amino acids (Gresner, Wichern, Lumpp, Hoedemaker, & Holtershinken, 2015). Plant‐mediated proteolysis generates peptides and amino acids (Beha et al., 2002). Microbial assimilation of amino acids precedes degradation to ammonia or BCVFA and use in microbial protein synthesis. Where fast plant‐mediated proteolysis occurs, there is an abundance of plant‐derived peptides and amino acids. We hypothesize that the lack of available energy to match availability of breakdown products favors deamination by hyperammonia‐producing bacteria leading to a rapid excess of ammonia in excess of capacity for microbial uptake (Figure 2). In contrast, where slow plant‐mediated proteolysis occurs there is less pressure to supply energy from the more limited supply of amino acids and more pressure on supply of components for microbial protein synthesis. We suggest that in this situation the balance of activity between hyperammonia‐producing bacteria and branched‐chain amino acid catabolism favors the latter to increase BCVFA relative to ammonia_._ This would also stimulate cellulolytic organisms to maintain protein conversion and microbial growth. We therefore propose that the limitation to protein incorporation and efficient feed use efficiency could be the relative proportions of BCVFA and ammonia (Figure 2).

Together, these observations suggest that the *Festulolium* hybrids evaluated in this experiment can promote substantial changes in rumen microbial fermentation and proteolysis with the potential to significantly improve (decrease) the environmental footprint of livestock agriculture. Full understanding of the significance and extent of this effect will entail an animal feeding experiment including measurements of N partitioning into urine and feces. Given the observations that the fermentative metabolism and colonizing rumen microbiome is affected by forage cultivar and species (Elliot et al., 2018; Kingston‐Smith, Marshall, et al., 2013), and the proposed role of BCVFA in promoting cellulolysis, we propose that the differences observed here relate to differential colonization of the *Festulolium* and *Lolium* grass cultivars by subsets of the microbiota within the rumen microbiome. This requires further investigation into the metagenomics of colonization of Festulolium as compared with the successional ecology established for *Lolium perenne* (Huws et al., 2016).

In conclusion, this work has demonstrated the potential for Festulolium to make a positive impact on efficiency and environmental impact of livestock farming. Festulolium varieties are commercially available although there is no tradition of growing them meaning that publicity is needed to increase uptake. At present, the Festulolium variety Aberniche (LmFg (4x)) is on the UK National and Recommended Lists, and AberLink a drought tolerant Italian with a Fg introgression is on the UK National List. The biggest challenge facing uptake of Festuloliums is the focus of the current testing system on yield regardless of additional quality considerations. There is no real category in which to test Festulolium, and so they tend to get placed either with the ryegrasses or hybrid ryegrass section. Finally, previous Festuloliums were of doubtful quality, thereby making them of limited interest to development from seed companies. However, the more recent listings of Aberniche and AberLink offer yields and quality that compare with ryegrass, bigger deeper root systems that help soil hydrology, soil stabilization, earthworm numbers and improve drought resistance MacLeod et al., 2013; Humphreys et al., 2018. Our results indicate that further hybrids should be developed with a focus on crosses between *L. perenne* and *F. marei* and *L. multiflorum* and *F. glaucescens*.

## CONFLICT OF INTEREST

The authors have declared that no competing interests exist.
